# High-quality-draft genome sequence of the multiple heavy metal resistant bacterium *Pseudaminobacter manganicus* JH-7^T^

**DOI:** 10.1186/s40793-018-0330-2

**Published:** 2018-10-25

**Authors:** Xian Xia, Jiahong Li, Zijie Zhou, Dan Wang, Jing Huang, Gejiao Wang

**Affiliations:** State Key Laboratory of Agricultural Microbiology, Huazhong Agricultural University, Wuhan, 430070 People’s Republic of China

**Keywords:** Cadmium, Exopolysaccharides, Heavy metal resistance and adsorption, Manganese,*Pseudaminobacter*

## Abstract

*Pseudaminobacter manganicus* JH-7^T^ (= KCTC 52258^T^ = CCTCC AB 2016107^T^) is a Gram-staining-negative, aerobic and non-motile strain that was isolated from a manganese mine*.* The strain JH-7^T^ shows multiple heavy metal resistance and can effectively remove Mn^2+^ and Cd^2+^. In addition, it is able to produce exopolysaccharides (EPS), which may contribute to metal remove/adsorption. Thus, strain JH-7^T^ shows a great potential in bioremediation of heavy metal-contaminated environment. In this study, we report the draft genomic sequence of *P. manganicus* JH-7^T^ and compare it to related genomes. Strain JH-7^T^ has a 4,842,937 bp genome size with a G + C content of 61.2%, containing 4504 protein-coding genes and 71 RNA genes. A large number of putative genes associated with heavy metal resistance and EPS synthesis are found in the genome.

**Electronic supplementary material:**

The online version of this article (10.1186/s40793-018-0330-2) contains supplementary material, which is available to authorized users.

## Introduction

Genus *Pseudaminobacter* was established by Kämpfer et al. in 1999 and contains three species represented by *Pseudaminobacter salicylatoxidans* BN12^T^ (type species) [1], *Pseudaminobacter defluvii* THI 051^T^ [1] and *Pseudaminobacter manganicus* JH-7^T^ [2]. The common characteristics of *Pseudaminobacter* strains are Gram-staining-negative, rod-shaped and aerobic [1, 2]. *P. salicylatoxidans* BN12^T^ contains a peculiar ring-fission dioxygenase with the ability to cleave salicylate in 1, 2-position to 2-oxohepta-3, 5-dienedioic acid [3].

*P. manganicus* JH-7^T^ was isolated from a sludge sample of a wastewater ditch in Dalong manganese mine in 2015 [2]. It shows multiple heavy metal resistance and can effectively remove Mn^2+^ and Cd^2+^. In addition, the strain produces EPS, which may facilitate heavy metal resistance and adsorption [4–6]. These features show great interests because of its potential applications in bioremediation of heavy metal contaminated environments. So far, only the genome of an atypical *Pseudaminobacter* strain *Pseudaminobacter salicylatoxidans* KCT001 has been sequenced [7]. Strain KCT001 can utilize tetrathionate as the substrate for sulfur-oxidizing chemolithotrophic growth [8]. For better understanding the mechanism of bacterial resistance and removal of heavy metals, here we analyze the genome of *P. manganicus* JH-7^T^.

## Organism information

### Classification and features

**Fig. 1:**
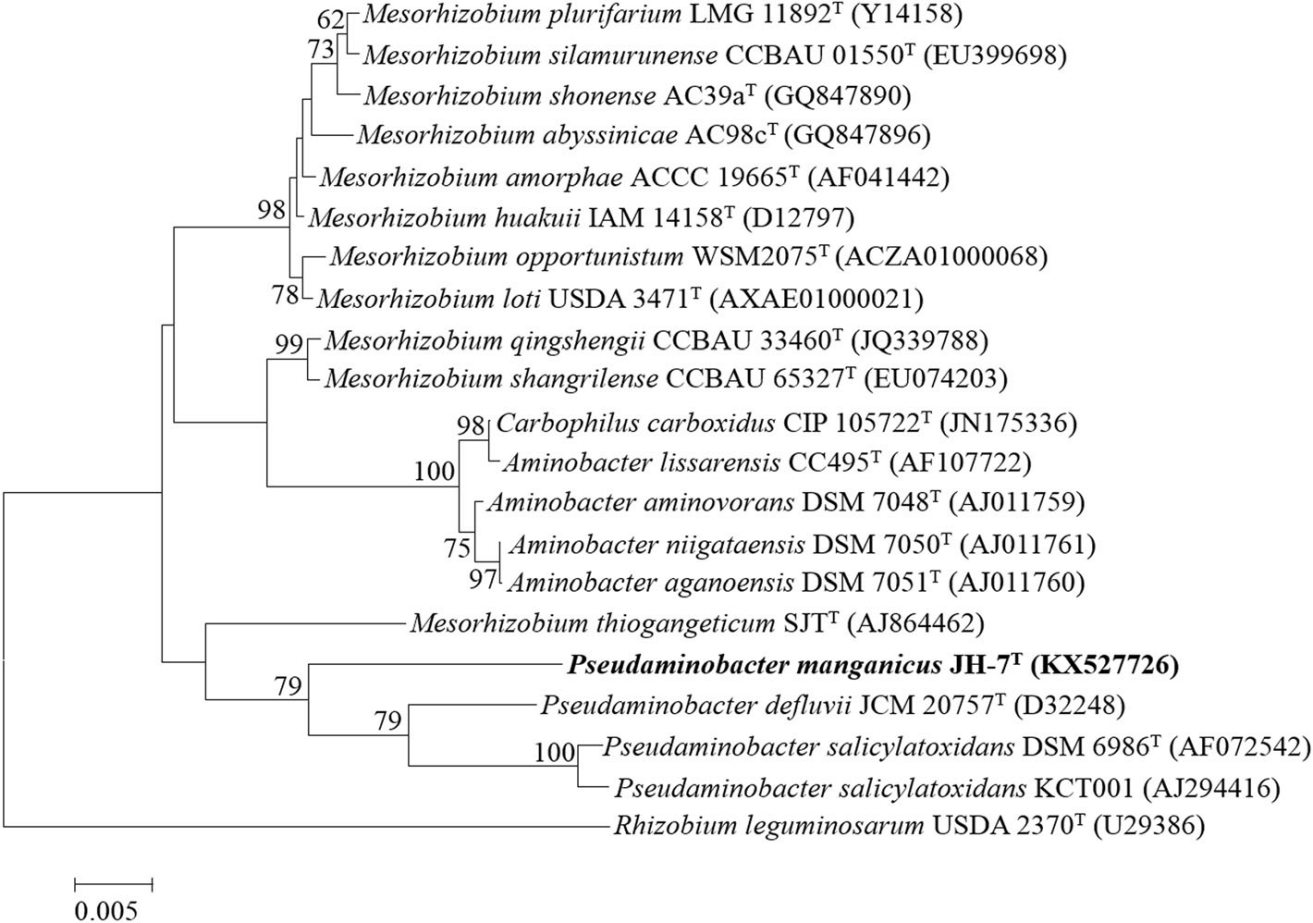
Phylogenetic tree highlighting the phylogenetic position of *Pseudaminobacter manganicus* JH-7^T^. The phylogenetic tree was constructed based on the 16S rRNA gene sequences. The analysis was inferred by MEGA 6.0 [41] with neighbor-joining algorithm and 1000 bootstrap repetitions were computed to estimate the reliability of the tree. Bar, 0.005 substitutions per nucleotide position

**Fig. 2:**
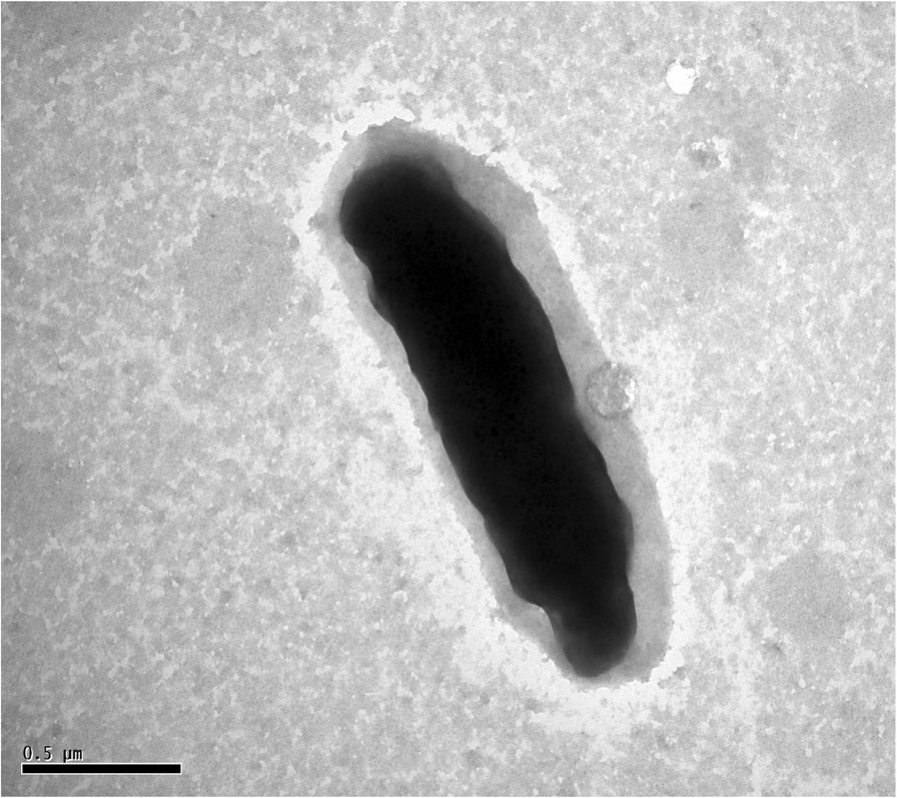
Transmission electron micrograph image of strain JH-7^T^. Bar, 0.5 μm

The phylogenetic relationship of *P. manganicus* JH-7^T^ to the related members is shown in a 16S rRNA gene based neighbor-joining tree. Strain JH-7^T^ is closely related to *P. salicylatoxidans* BN12^T^, *P. defluvii* THI 051^T^ and *P. salicylatoxidans* KCT001 (Fig. 1). Strain JH-7^T^ is Gram-staining-negative, aerobic, non-motile and rod-shaped (0.3–0.8 × 1–2 μm) (Fig. 2). The colonies are white, circular, entire, slightly raised and smooth on LB agar plates. It is positive for oxidase and catalase activities and hydrolysis of casein [2]. The major fatty acids are C_18:1_ *ω*7*c*, C_19:0_ cyclo *ω*8*c* and C_16:0_ and the G + C content is 61.2 mol% [2]. The major polyamine is sym-homospermidine and the respiratory quinone is ubiquinone-10. The polar lipids are phosphatidylmonomethylethanolamine, diphosphatidylglycerol, phosphatidylglycerol, phosphatidylcholine, two aminolipids and two lipids [2]. Table 1 shows the general features of *P. manganicus* JH-7^T^.

**Table 1:** Classification and general features of *P. manganicus* JH-7^T^ [42]

MIGS ID	Property	Term	Evidence code^a^
	Classification	Domain *Bacteria*	TAS [43]
		Phylum *Proteobacteria*	TAS [44, 45]
		Class *Alphaproteobacteria*	TAS [46]
		Order *Rhizobiales*	TAS [46, 47]
		Family *Phyllobacteriaceae*	TAS [46, 47]
		Genus *Pseudaminobacter*	TAS [1, 2]
		Species *manganicus*	TAS [2]
		Type strain JH-7^T^ (= KCTC 52258^T^ = CCTCC AB 2016107^T^)	TAS [2]
	Gram stain	negative	TAS [2]
	Cell shape	rod-shaped	TAS [2]
	Motility	no	TAS [2]
	Sporulation	no	TAS [2]
	Temperature range	15–40 °C	TAS [2]
	Optimum temperature	28 °C	TAS [2]
	pH range; Optimum	5–9; 7	TAS [2]
	Carbon source	D-glucose, L-arabinose, D-fructose and D-mannose	TAS [2]
MIGS-6	Habitat	Mine sludge	TAS [2]
MIGS-6.3	Salinity	0–6% NaCl (*w*/*v*)	TAS [2]
MIGS-22	Oxygen requirement	aerobic	TAS [2]
MIGS-15	Biotic relationship	free-living	TAS [2]
MIGS-14	Pathogenicity	non-pathogen	NAS
MIGS-4	Geographic location	Tongren city, Guizhou province, P. R. China	TAS [2]
MIGS-5	Sample collection	2015	TAS [2]
MIGS-4.1	Latitude	N27° 43′ 8"	TAS [2]
MIGS-4.2	Longitude	E108° 31′ 42"	TAS [2]
MIGS-4.4	Altitude	not reported	

These evidence codes are from the Gene Ontology project [48]

*IDA* Inferred from Direct Assay, *TAS* Traceable Author Statement (i.e., a direct report exists in the literature), *NAS* Non-traceable Author Statement (i.e., not directly observed for the living, isolated sample, but based on a generally accepted property for the species, or anecdotal evidence)

^a^Evidence codes

**Fig. 3:**
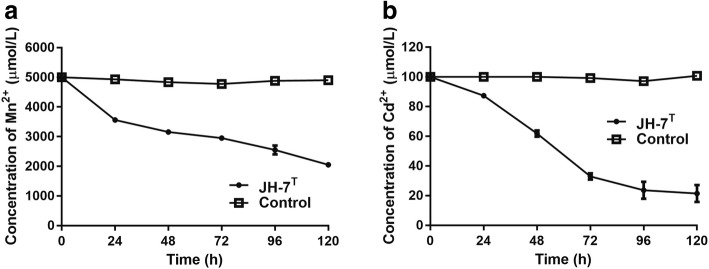
Mn^2+^ and Cd^2+^ removed by *P. manganicus* JH-7^T^. Control stands for null LB medium. Strain JH-7^T^ was incubated until OD_600_ reach 1.0, and then amended with 5000 μmol/L MnCl_2_ (**a**) and 100 μmol/L CdCl_2_ (**b**), respectively. The cultures were removed at 24 h intervals. After centrifuging at 12,000 rpm for 10 min, the supernatant was used to determine the residual concentration of Mn^2+^ and Cd^2+^ by the atomic absorption spectrometry AAS (AAS; 986A, Beijing Puxi General Instrument 197 Co., Beijing, China). Bars represent the mean ± SD of three biological replicates

The resistant levels of *P. manganicus* JH-7^T^ to multiple metal(loid)s were tested with the MIC on LB agar plates incubated at 28 °C for 7 days. The MICs for MnCl_2_, CdCl_2,_ PbCl_2_, CuCl_2_, ZnSO_4_ and NiSO_4_ are100, 2, 10, 5, 5 and 5 mmol/L respectively. The MICs for K_2_CrO_4_ and Na_3_AsO_3_ are both 0.1 mmol/L that are lower than the above six metals. Specifically, strain JH-7^T^ could remove nearly 60% of 5 mmol/L Mn^2+^ and nearly 80% of 0.1 mmol/L Cd^2+^ (Fig. 3), respectively. In addition, strain JH-7^T^ could produce EPS based on the aniline blue reaction incubated on LB agar in 3–7 days [9] (data not shown). This phenomenon is consistent with the cell image observed by TEM (Fig. 2). A lay of shadow around the strain was similar to the EPS observed in strain *Bifidobacterium longum* 35,624 [10].

## Genome sequencing information

### Genome project history

This organism was selected for sequencing particularly due to its multiple heavy metals resistance and heavy metal removal ability. Genome sequencing was performed by Wuhan Bio-Broad Co., Ltd., Wuhan, China in 2016. The draft genome sequence of strain *P. manganicus* JH-7^T^ has been deposited at DDBJ/EMBL/GenBank under accession number MDET00000000. The project information is summarized in Table 2.

**Table 2:** Project information

MIGS ID	Property	Term
MIGS-31	Finishing quality	High-quality draft
MIGS-28	Libraries used	Illumina Paired-End library (300 bp insert size)
MIGS-29	Sequencing platforms	Illumina Miseq 2000
MIGS-31.2	Fold coverage	624.94×
MIGS-30	Assemblers	SOAPdenovo v2.04
MIGS-32	Gene calling method	GeneMarkS^+^
	Locus TAG	BFN67
	Genbank ID	MDET00000000
	Genbank Date of Release	31, March, 2017
	GOLD ID	Gp0291525
	Bioproject	PRJNA338732
MIGS-13	Source material identifier	CCTCC AB 2016107^T^
	Project relevance	Bioremediation

### Growth conditions and genomic DNA preparation

*P. manganicus* JH-7^T^ was grown under aerobic conditions in LB medium at 28 °C for 40 h. DNA extraction was performed using the QiAamp kit (Qiagen, Germany) as the manufacturer’s instructions. A NanoDrop Spectrophotometer 2000 was used to determine the quality and quantity of the DNA. Seven microgram of DNA was sent to Bio-broad Technogoly Co., Ltd., Wuhan, China for sequencing.

### Genome sequencing and assembly

The genome of strain JH-7^T^ was sequenced on Illumina Hiseq2000 [11] and assembled by Bio-broad Technogoly Co., Ltd., Wuhan using SOAPdenovo v2.04 [12]. An Illumina standard shotgun library was constructed and sequenced, which generated 19,404,755 reads totaling 2,885,684,230 bp and average of 625 times genome coverage. The total size of the genome is 4,842,937 bp and a total of 60 scaffolds were obtained after arranging 68 contigs together. The part gaps of assembly were filled and the error bases were revised using GapCloser v1.12 [13].

### Genome annotation

The draft genome was annotated through the NCBI Prokaryotic Genome Annotation Pipeline (PGAP), and genes were identified using the gene caller GeneMarkS^+^ with the similarity-based gene detection approach [14]. The predicted CDSs were translated and were submitted to the Pfam protein family database [15] and KEGG database [16]. The genes in internal clusters were performed by OrthoMCL [17, 18]. The protein function classification, transmembrane helices and signal peptides were predicted by WebMGA [19], TMHMM v. 2.0 [20] and SignalP 4.1 [21], respectively. In addition, the CRISPRfinder program [22] was used to predict CRISPRs in the genome.

## Genome properties

The draft genome size of strain JH-7^T^ is 4,842,937 bp with 61.2 mol% G + C content and contains 60 scaffolds. The genome properties and statistics are shown in Table 3. From a total of 4685 genes, 4504 (96.2%) are protein coding genes, 110 (2.3%) are pseudo genes and the rest are 71 predicted RNA genes, including 54 tRNA, 12 rRNAs and 5 ncRNA. In addition, 3729 (82.8%) protein coding genes are distributed into COG functional categories (Table 4).

**Table 3:** Genome statistics

Attribute	Value	% of total^a^
Genome size (bp)	4,842,937	100
DNA coding (bp)	4,238,496	87.5
DNA G + C (bp)	2,963,726	61.2
DNA scaffolds	60	100
Total genes^b^	4685	100
Protein-coding genes	4504	96.2
RNA genes	71	1.7
Pseudo genes	110	2.3
Genes in internal clusters	1725	38.3
Genes with function prediction	3228	68.9
Genes assigned to COGs	3729	79.6
Genes with Pfam domains	3926	83.8
Genes with signal peptides	392	8.4
Genes with transmembrane helices	1119	23.9
CRISPR repeats	5	

^a^The total is based on either the size of the genome in base pairs or the total number of protein coding genes in the annotated genome

^b^Also includes 110 pseudogenes, 54 tRNA genes, 12 rRNAs and 5 ncRNA

**Table 4:** Number of genes associated with the 25 general COG functional categories

Code	Value	% of total^a^	Description
J	181	4.02	Translation
A	0	0.00	RNA processing and modification
K	299	6.64	Transcription
L	233	5.17	Replication, recombination and repair
B	3	0.07	Chromatin structure and dynamics
D	39	0.87	Cell cycle control, mitosis and meiosis
Y	0	0.00	Nuclear structure
V	46	1.02	Defense mechanisms
T	134	2.98	Signal transduction mechanisms
M	217	4.82	Cell wall/membrane biogenesis
N	35	0.78	Cell motility
Z	0	0.00	Cytoskeleton
W	0	0.00	Extracellular structures
U	106	2.35	Intracellular trafficking and secretion
O	156	3.46	Posttranslational modification, protein turnover, chaperones
C	240	5.33	Energy production and conversion
G	312	6.93	Carbohydrate transport and metabolism
E	482	10.70	Amino acid transport and metabolism
F	87	1.93	Nucleotide transport and metabolism
H	158	3.51	Coenzyme transport and metabolism
I	153	3.40	Lipid transport and metabolism
P	209	4.64	Inorganic ion transport and metabolism
Q	91	2.02	Secondary metabolites biosynthesis, transport and catabolism
R	453	10.06	General function prediction only
S	444	9.86	Function unknown
–	775	17.21	Not in COGs

^a^The total is based on the total number of protein coding genes in the annotated genome

## Insights from the genome sequence

Strain JH-7^T^ could tolerant multiple heavy metals (Mn^2+^, Cd^2+^_,_ Pb^2+^, Cu^2+^, Zn^2+^ and Ni^2+^) and remove Mn^2+^ and Cd^2+^, suggesting that it has developed a number of evolutionary strategies to adapt the mine environment. According to the genome annotation results, strain JH-7^T^ harbors various putative proteins related to heavy metal(loid)s resistance including transporters, resistance proteins and metal reductases (Additional file 1: Table S1). MntH [23] and metal ABC transport system [24] are involved in cation uptake. Heavy metal-transporting ATPase is responsible for the efflux of Pb^2+^, Zn^2+^, Cd^2+^ and Ni^2+^ [25–28]. The genome contains Cu^2+^ efflux system CopABC [29], mercuric reductase MerA and regulator MerR [30]. Athough the MICs for Cr^6+^ and As^3+^ are not high, the Cr^6+^ efflux protein ChrA [27, 31] and As^3+^ resistant proteins (ArsRHC and ACR3) [32–34] are present.

**Fig. 4:**
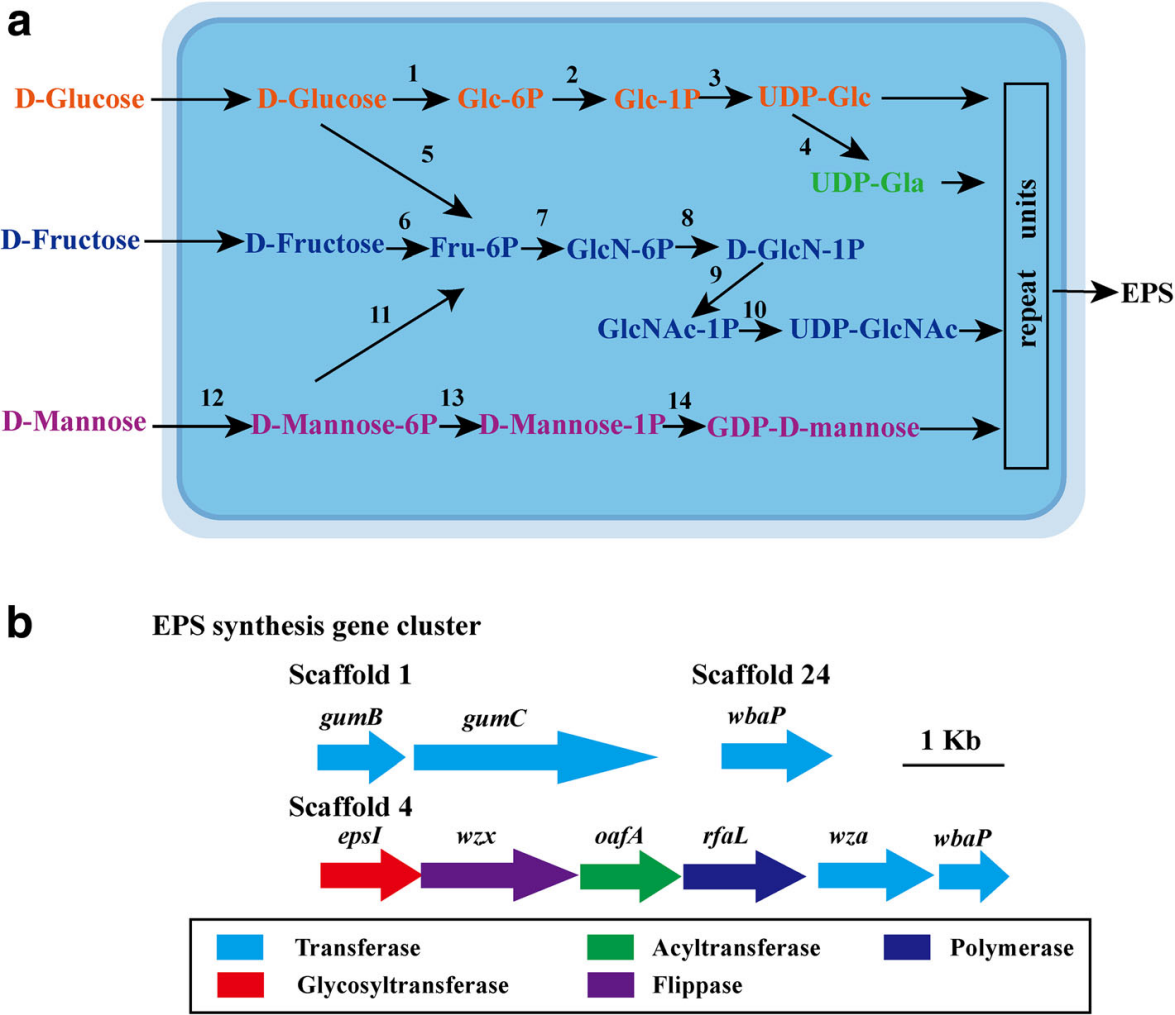
Putative nucleotide sugars biosynthesis pathway and EPS synthesis gens in *P. manganicus* JH-7^T^. **a** The predicted nucleotide sugars biosynthesis pathway. The numbers refer to the enzymes involved: 1, Glucokinase; 2, α-D-glucose phosphate-specific phosphoglucomutase; 3, UTP--glucose-1-phosphate uridylyltransferase; 4, UDP-glucose 4-epimerase GalE; 5, Glucose-6-phosphate isomerase; 6, Fructokinase; 7, Glutamine--fructose-6-phosphate aminotransferase; 8, Phosphoglucosamine mutase; 9, UDP-N-acetylglucosamine; 10, Glucose-6-phosphate isomerase; 11, Mannose-6-phosphate isomerase; 12, PTS-Man-EIIA, ManX; 13, Phosphoglucomutase; 14, Mannose-1-phosphate guanylyltransferase. **b** The EPS synthesis gene cluster in strain JH-7^T^

EPS are long-chain polysaccharides consisting of branched, repeating units of sugars or sugar derivatives [35]. Stain JH-7^T^ could produce EPS and all essential proteins for EPS production are found in the genome. Four complete nucleotide sugar synthesis (EPS precursor) pathways are identified based on KEGG analysis (Additional file 1: Table S2) including the syntheses of UDP-glucose, UDP-galactose, UDP-GlcNAc and GDP-D-mannose (Fig. 4a). EPS assembly gene clusters were also found in the genome of strain JH-7^T^ [36] (Additional file 1: Table S3, Fig. 4b). Based on gene analysis, it is suggested that the EPS assembly in strain JH-7^T^ might belong to Wzx/Wzy-dependent pathway [37], e.g., repeat units are assembled by glycosyltransferases (EpsI) and translocated across the cytoplasmic membrane to periplasm by flippase (Wzx) [37] and WbaP [38]. Next, Wzy (RfaL), polysaccharide co-polymerase (GumC) and the outer membrane polysaccharide exporter (GumB) transports the polymerized repeat units to cell surface [37, 39]. EPS has been reported to contribute to heavy metal removal/adsorption in bacteria [3–6]. Hence, the ability of EPS may contribute to Mn^2+^ and Cd^2+^ removal.

**Fig. 5:**
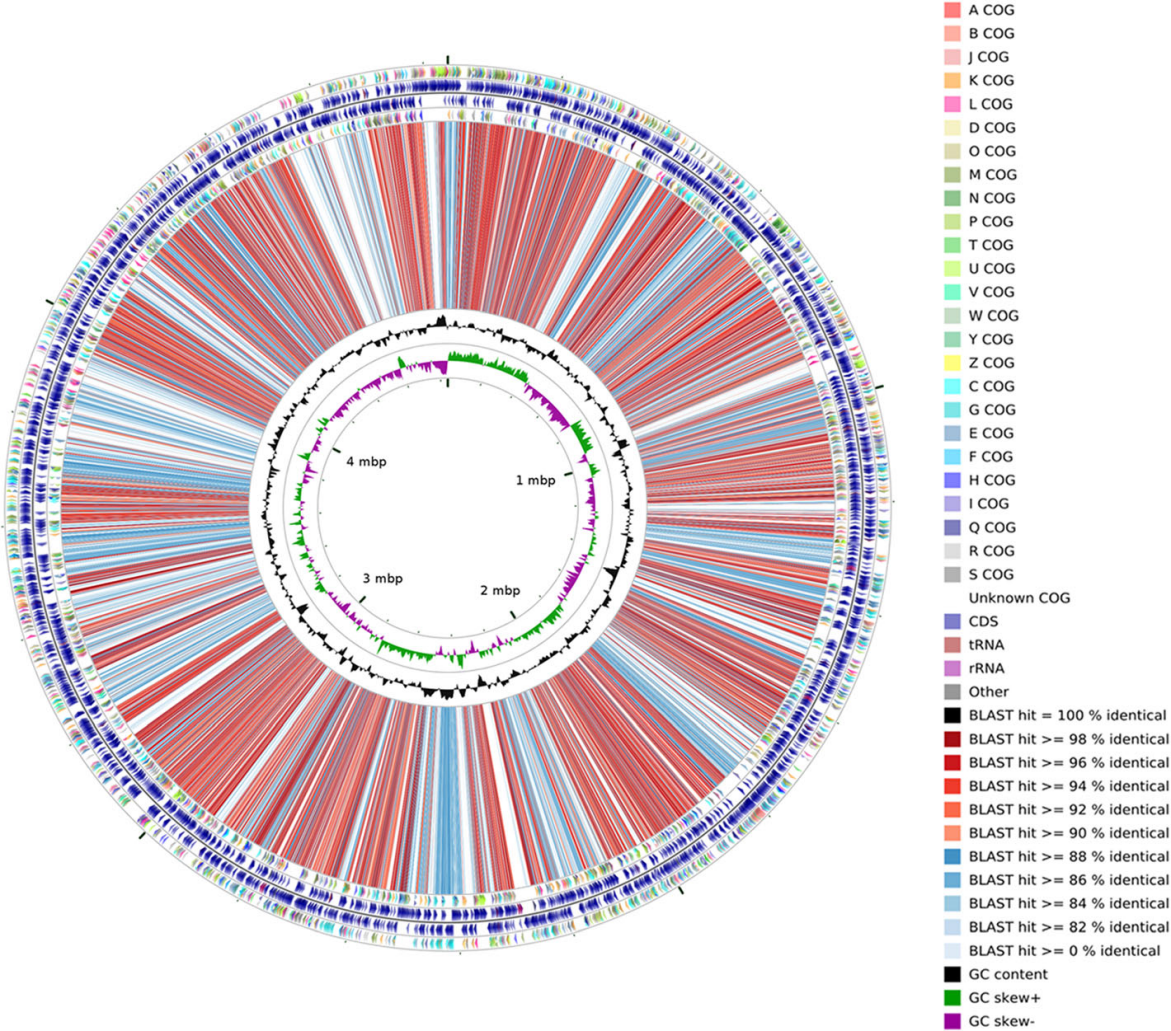
A graphical circular map of the comparison between strain *P. manganicus* JH-7^T^ and *P. salicylatoxidans* KCT001. From outside to center, rings 1, 4 show protein-coding genes colored by COG categories on forward/reverse strand; rings 2, 3 denote genes on forward/reverse strand; rings 5 show the CDS vs CDS BLAST results of strain JH-7^T^ with strain KCT001; ring 6 shows G + C % content plot and the innermost ring shows GC skew

To gain more insight, the genomic features of strain JH-7^T^ is compared with the available genome *P. salicylatoxidans* KCT001 [7]. Strain JH-7^T^ has similar genome size (4.84 Mbp) and G + C content (61.2 mol%) compared to strain KCT001 (4.61 Mbp; 62.8 mol%). A total of 2408 core proteins are shared between the two strains. Strain JH-7^T^ has 1724 strain-specific CDSs. Figure 5 shows the genome comparison results of strain JH-7^T^ and strain KCT001 using CGview comparison tool [40]. Comparing to *P. salicylatoxidans* KCT001, strain JH-7^T^ was unable to utilize tetrathionate for chemolithoautotrophy (data not shown). However, it harbors high quantitative and diverse heavy metal resistance genes.

## Conclusions

To the best of our knowledge, this study provides the first typical strain genomic information of the genus *Pseudaminobacter* and revealed a consistency of important characters between genotypes and phenotypes. Strain JH-7^T^ is resistant to multiple heavy metals and capable of removal Mn^2+^/Cd^2+^. Genome analysis reveal various genes responsible for multiple heavy metal resistance, which provides the genomic basis for this strain to adapt the harmful environment.

## Additional file

Additional file 1: **Table S1.** Putative heavy metal(loid)s resistance proteins. **Table S2.** Putative nucleotide sugars biosynthesis proteins for EPS production. **Table S3.** Putative proteins for EPS production. (XLSX 11 kb)

## Abbreviations

EPS: Exopolysaccharides
MIC: Minimal inhibition concentration
